# Structural and Enzymatic characterization of the lactonase *Sis*Lac from *Sulfolobus islandicus*

**DOI:** 10.1371/journal.pone.0047028

**Published:** 2012-10-10

**Authors:** Julien Hiblot, Guillaume Gotthard, Eric Chabriere, Mikael Elias

**Affiliations:** 1 URMITE UMR CNRS-IRD 6236, IFR48, Faculté de Médecine et de Pharmacie, Université de la Méditerranée, Marseille, France; 2 Weizmann Institute of Science, Biological Chemistry, Rehovot, Israel; University Paris Diderot-Paris 7, France

## Abstract

**Background:**

A new member of the Phosphotriesterase-Like Lactonases (PLL) family from the hyperthermophilic archeon *Sulfolobus islandicus* (*Sis*Lac) has been characterized. *Sis*Lac is a native lactonase that exhibits a high promiscuous phosphotriesterase activity. *Sis*Lac thus represents a promising target for engineering studies, exhibiting both detoxification and bacterial *quorum* quenching abilities, including human pathogens such as *Pseudomonas aeruginosa*.

**Methodology/Principal Findings:**

Here, we describe the substrate specificity of *Sis*Lac, providing extensive kinetic studies performed with various phosphotriesters, esters, *N*-acyl-homoserine lactones (AHLs) and other lactones as substrates. Moreover, we solved the X-ray structure of *Sis*Lac and structural comparisons with the closely related *Sso*Pox structure highlighted differences in the surface salt bridge network and the dimerization interface. *Sis*Lac and *Sso*Pox being close homologues (91% sequence identity), we undertook a mutational study to decipher these structural differences and their putative consequences on the stability and the catalytic properties of these proteins.

**Conclusions/Significance:**

We show that *Sis*Lac is a very proficient lactonase against aroma lactones and AHLs as substrates. Hence, data herein emphasize the potential role of *Sis*Lac as *quorum* quenching agent in *Sulfolobus*. Moreover, despite the very high sequence homology with *Sso*Pox, we highlight key epistatic substitutions that influence the enzyme stability and activity.

## Introduction

*Sis*Lac (also known as *Sis*Pox [1]) is an enzyme isolated from the archaeon organism *Sulfolobus islandicus*, which is found in extreme environments like the Yellowstone natural park (U.S.A.) or the Mutnovksy volcano in Kamchatka (Russia) [2]. *Sis*Lac belongs to an enzyme family called Phosphotriesterase-Like Lactonase (PLL) that encompasses members from mesophilic organisms (PPH, AhlA, MCP, *Dr*OPH) [3], [4], [5] as well as thermophilic (*Sso*Pox, *Sac*Pox, *Gs*P, *Gk*L) [6], [7], [8], [9] representatives. The PLL family is structurally and biochemically related to bacterial Phosphotriesterases (PTEs) [3]. Indeed, some representatives of PLLs were primarily isolated by virtue of their phosphotriesterase activity towards the insecticide paraoxon, and were named paraoxonases (Pox) as was the case with *Sis*Lac’s closest homologue, *Sso*Pox, an enzyme isolated from *Sulfolobus solfataricus* [6]. However, further phylogenetic and biochemical studies has revealed that these enzymes are native lactonases endowed with promiscuous paraoxonase activity and more generally with organophosphate hydrolase activity [3]. Hyperthermophylic PLLs (hPLLs) are appealing enzymes in biotechnology because they possess an intrinsically high stability that often confer high resistance towards harsh conditions and proteases activity [10], which constitute useful properties for storage and large scale purification.

Interestingly, PTEs exhibit diffusion limit-like second order rates with paraoxon as a substrate [11], and are also endowed with promiscuous lactonase activity [3], [12]. The particular link between these two families raises the hypothesis that PTEs have diverged from native lactonases like PLLs [3], [13], [14], [15]. Indeed, both PTEs and PLLs belong to the amidohydrolase superfamily [3]. Despite the relatively low sequence identity between these two families (∼30%), PTEs and PLLs exhibit the same (β/α)_8_ barrel fold or so-called TIM barrel [16]. At the C-terminus of the barrel, two divalent metal cations constitute the active site [13], [17], [18]. The phosphotriesterase activity of PTEs and PLLs is modulated in the presence of various divalent cations, the highest activity being achieved in a cobalt-containing buffer for *Pseudomonas diminuta* PTE [19], OpdA [20] and *Sso*Pox [6].The active site metal cations’ chemical nature has been investigated using anomalous X-ray scattering and has revealed that both the PTE from *Agrobacterium radiobacter* (OpdA) and the PLL *Sso*Pox possess an iron/cobalt heterobinuclear center when the expression media is supplemented with cobalt ions [13], [20].

The catalytic mechanisms for both the lactonase and phosphotriesterase involve a nucleophilic attack by a water molecule activated by the bi-metallic center. The major difference between the two activities consists of the respective transition state geometries: bi-pyramidal for the phosphotriesters and tetrahedral for the lactones. The fact that these two activities can be catalyzed with significant rates within the same active site suggests an overlap between the stabilization of the corresponding transitions state species, from which the enzymatic promiscuity would stem from [14], [21]. The active site of PLLs possesses three sub-sites that are remarkably adapted for the lactone binding: a small sub-site, a large sub-site and a hydrophobic channel [13]. The aliphatic chain of the lactones binds within the hydrophobic channel, the large sub-site accommodates the amide group of the *N*-acyl chain, and the small sub-site positions the lactone ring. AHLs are molecules that mediate bacterial communication (*quorum* sensing) for many species. These molecules accumulate in the media to reach a certain threshold for which the transcriptional profile of the bacteria is altered [22]. Some studies report that virulence and biofilm life-style are regulated by *quorum* sensing [23], [24], [25], [26], and suggest that quenching the *quorum* sensing (*quorum* quenching) could be an interesting strategy against multi-resistant pathogen bacteria using AHL based *quorum* sensing like *P. aeruginosa* [27], [28], [29], [30], [31]. By hydrolyzing AHLs, lactonases like PLLs can quench the AHL-mediated communication between bacteria, as seen for AiiA [28] or for human paraoxonases [30].

Because of their dual catalytic activities, lactonases and phosphotriesterases, PLLs constitute highly attractive candidate for biotechnological utilization as *quorum* quenching agent [32] or OPs biodecontaminant [33]. However, their precise biological function(s) remain(s) unknown. In many cases, PLLs are found in bacteria that do not produce or sense AHLs, which may suggest a role in interfering with the *quorum* sensing of other organisms or in the metabolism of the AHL molecules. Furthermore, some members of the PLL family efficiently hydrolyze gamma and/or delta oxo-lactones, but not AHLs [9], [34]. However, the structural features that determine the lactonase specificity of these two classes remain unknown.

In this work we present a structural and biochemical analysis of *Sis*Lac. We have performed a detailed kinetic characterization and show that this enzyme hydrolyzes AHLs, but also γ and δ-lactones with high proficiency. Moreover, we provide a characterization of its thermal stability, thermophilicity and structural determinants responsible of its stability. The structural comparison with *Sso*Pox reveals an important dimer interface change. Key amino acids variations between the two close homologues were analyzed by mutagenesis study and revealed their critical involvement in the protein stability and lactonase activity.

## Materials and Methods

### Strain, Plasmids and Site Directed Mutagenesis

The plasmids preparations were performed in *Escherichia coli* strain DH5α (Invitrogen). Protein production was performed in *E. coli* BL21(DE_3_)-pGro7/GroEL strain (TaKaRa) using plasmids pET22b-StrepTev*Sis*Lac and pET22b-*Sso*Pox (provided by GeneArt; Germany). Site directed mutagenesis was performed in 50 µL using *Pfu* polymerase (Invitrogen) on 100 ng of plasmid encoding corresponding genes and primers referenced in **Table S1**. The PCR cycle was performed using hybridization temperature of 55°C, elongation time of 12 min during 30 cycles and final elongation of 20 min. The template plasmid was eliminated by Fast Digest *Dpn*I (Fermentas) digestion of 30 min at 37°C followed by inactivation step of 20 min at 80°C. Plasmids were concentrated by classical alcoholic precipitation and then electroporated (Gene-Pulser, Bio-Rad) into *E. cloni 5alpha* cells (Lucingen), a particularly competent strain of *E. coli*. Site directed mutagenesis was finally verified by sequencing.

### Production-purification of SisLac, SsoPox and SisLac’s Variants

*Sis*Lac and its variants were heterologously produced and purified from the *Escherichia coli* strain BL21(DE3)-pGro7/GroEL (TaKaRa) as previously described [1] with the only difference being that 0.2% (w/v) arabinose was added at the start of the over-expression in order to induce chaperones expression. A very similar production protocol was used for *wt Sso*Pox. Briefly, protein production was performed in 2 liters of ZYP medium [35] (100 µg/ml ampicillin, 34 µg/ml chloramphenicol) inoculated by over-night pre-culture at a 1/20 ratio. Cultures grew at 37°C to reach OD_600nm_ = 1.5. The induction of protein production was made by starting the consumption of the lactose in ZYP medium. Subsequently, 0.2 mM CoCl_2_ was added and the temperature was reduced to 25°C for additional 20 hours. Cells were harvested by centrifugation (3 000 g, 4°C, 10 min), re-suspended in lysis buffer (50 mM HEPES pH 8, 150 mM NaCl, CoCl_2_ 0.2 mM, Lysozyme 0.25 mg/ml, PMSF 0.1 mM DNAseI 10 µg/ml) and stored at −80°C. The frozen cells were thawed and disrupted by three steps of 30 seconds of sonication (Branson Sonifier 450; 80% intensity and microtype limit of 8). Cell debris were removed by centrifugation (12 000 g, 4°C, 30 min). The purified protein being hyperthermostable, host proteins were precipitated by incubation of 30 min at 70°C and harvested by centrifugation (12 000 g, 4°C, 30 min). Other thermostable proteins from the host *E. coli* were eliminated by ammonium sulfate precipitation (326 g/L), and the overexpressed protein was concentrated by ammonium sulfate precipitation (476 g/L) and suspended in *activity buffer* (HEPES 50 mM pH 8, NaCl 150 mM, CoCl_2_ 0,2 mM). The remaining ammonium sulfate was removed by dialysis against the *activity buffer* and the protein sample was subsequently concentrated prior to the size exclusion chromatography step (S75-16-60, GE Healthcare). The yield of protein production varied between 20 and 100 mg of protein per liter of culture after purification. The purity and the protein quality were verified by SDS-PAGE and mass spectrometry.

### Oligomerization State Determination

*Dynamic light scattering (DLS)*: experiments were performed at room temperature using zetasizer nano series apparatus (Malvern, UK) and the Zetasizer software. 30 µL of purified *wt Sso*Pox and *Sis*Lac (2.5 mg/mL) was used in the *activity buffer* to measure the hydrodynamic radius of particles in the protein solutions at 633 nm.

*Multi-angle Light Scattering Studies*: experiments were performed at room temperature using a size exclusion chromatography (KW803 column (Shodex)) carried out on an Alliance 2695 HPLC system (Waters) at a flow rate of 0.5 ml/min. The buffer used was similar to *activity buffer* despite pH was adjusted to 7.3 using NaOH. The signal was monitored by a three-angle light-scattering detector (MiniDAWNTMTREOS; Wyatt Technology), a quasi-elastic light-scattering instrument (DynaproTM, Wyatt Technology), and a differential refractometer (Optilab rEX, Wyatt Technology). Molecular weight, gyration, and hydrodynamic radii determination were performed by the ASTRA V software (Wyatt Technology) using a dn/dC (specific refractive index increment of the solution) value of 0.185 ml/g.

### Enzymatic Characterization

The time course of ethyl-paraoxon hydrolysis by *Sis*Lac at 70°C was monitored following the *p*-nitrophenolate production at 405 nm (ε_405nm_ = 17 000 M^−1^cm^−1^) in 1-cm path length cell with a Cary WinUV spectrophotometer (Varian, Australia) and using the Cary WinUV software. Standard assays (500 µL) were performed in *paraoxonase buffer* CHES 50 mM pH 9, NaCl 150 mM, CoCl_2_ 0.2 mM, EtOH 6% (v/v), with pH adjusted with NaOH at 70°C.

**Figure 1:**
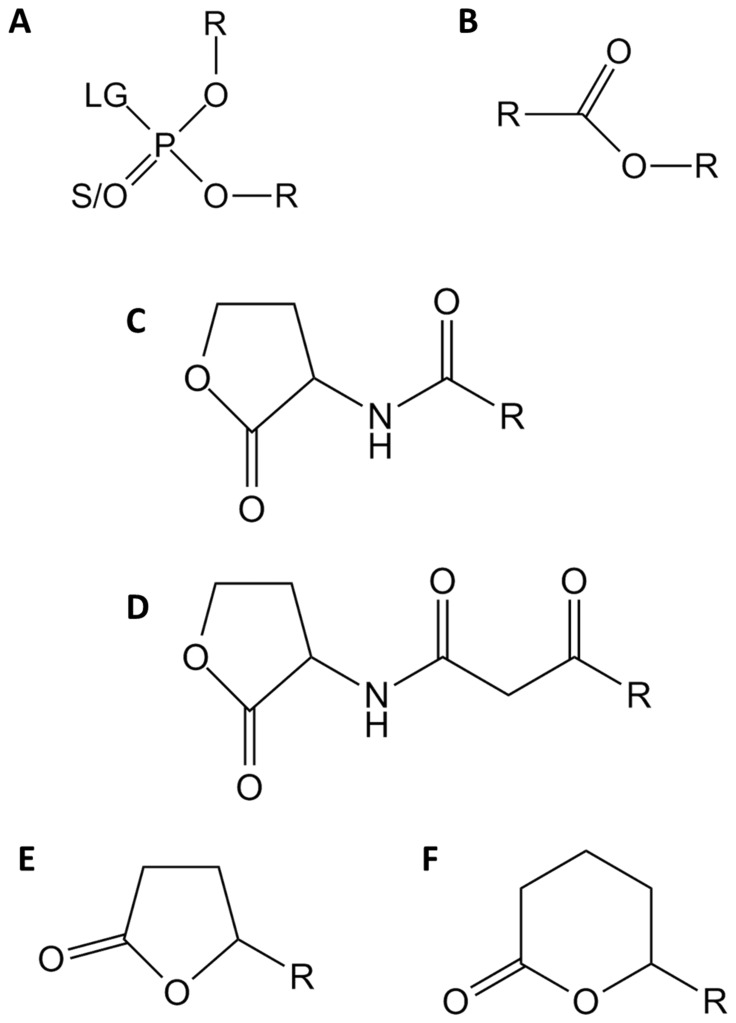
Chemical structure of *Sis*Lac substrates. Chemical structures of (**A.**) phosphotriesters, (**B.**) esters, (**C.**) Acyl-Homoserine Lactones, (**D.**) 3-oxo-Acyl-Homoserine Lactones (**E.**) γ-lactones and (**F.**) δ-lactones are presented. For phosphotriesters, R corresponds to different nature of substituents, LG corresponds to the leaving group which can be F, S-R, O-R or CN. The terminal substituent could be S atom if the molecule is a thionophosphotriester or an O atom if the molecule is an oxonphosphotriester. For esters, R corresponds to different nature of substituent. For AHLs and γ/δ-lactones, R corresponds to different size of acyl chain.

At 25°C, the phosphotriesterase, esterase and lactonase activities were analyzed monitoring absorbance variations in 200 µL reaction volumes using 96-well plates (6.2-mm path length cell) and a microplate reader (Synergy HT) using the Gen5.1 software at 25°C. For each substrate, assays were performed using organic solvent concentrations below 1%. The monitoring wavelength, the solvent used, the molar extinction coefficient and the concentration range for each substrate (**Fig. 1** **, S1 & S2**) are summarized in **Table S2**. Phosphotriesterase and esterase activities were performed in *activity buffer*. When required, DTNB at 2 mM was added to the buffer to follow hydrolysis of substrate releasing thiolate group (malathion (**Fig. S1V**)). Catalytic parameters for some phosphotriesters were also recorded using SDS at concentrations 0.01 and 0.1% (w/v). Lactone hydrolysis assays were performed in *lactonase buffer* (Bicine 2.5 mM pH 8.3, NaCl 150 mM, CoCl_2_ 0.2 mM, Cresol purple 0.25 mM and 0.5% DMSO) using cresol purple (pK_a_ 8.3 at 25°C) as pH indicator to follow the acidification related to the lactone ring hydrolysis. Molar coefficient extinction was measured by recording absorbance of the buffer over a range of acetic acid concentrations (0–0.35 mM). The absorbance values *versus* acetic acid concentration were fitted to a linear regression (GraphPad Prism 5 software) with a slope corresponding to molar extinction coefficient (see **Table S2**). For all experiments, each point was made in triplicate and the Gen5.1 software was used to evaluate the initial velocity at each substrate concentration. Mean values were fitted to the Michaelis-Menten equation using Graph-Pad Prism 5 software to obtain the catalytic parameters. In the case of C4 AHL hydrolysis for which the substrate concentration that enable to determine the enzyme V_max_ could not be reached, the catalytic efficiency has been determined by fitting the linear part of the Michaelis-Menten plot to a linear regression.

### Thermostability Analysis

#### Temperature dependence analysis

The temperature dependence of the *Sis*Lac paraoxonase activity was studied over the temperature range 25–85°C with 10°C increment. The ethyl-paraoxon (2 mM) hydrolysis was monitored in 500 µL at 405 nm (ε_405nm_ = 17 000 M^−1^cm^−1^) in 1-cm path length cell with a Cary WinUV spectrophotometer (Varian, Australia) using the Cary WinUV software. Triplicate experiments were performed in *paraoxonase buffer* with pH adjusted with NaOH to 9 at each temperature.

#### Activity-based thermal stability

The residual paraoxonase activity of *Sis*Lac after incubation at different temperatures was performed. Incubation time and temperatures tested in this experiment were 390 min at 85°C, 90°C and 95°C in *activity buffer*. The ethyl-paraoxonase (1 mM) activity was followed at 25°C every 15 min during the first hour of incubation and every 30 min till the end of the experiment (after cooling). Paraoxon hydrolysis was monitored at 405 nm (ε_405nm_ = 17 000 M^−1^cm^−1^) with a microplate reader (Synergy HT) and Gen5.1 software in a 6.2 mm path length cell for 200 µL reaction in 96-well plate. Initial velocity at 25°C was used as reference to normalize velocities obtained after incubation at high temperature. Values are represented as fraction of the reference (value 1.0). The half-life of the protein at each temperature was determined by fitting the data to an exponential decay equation using GraphPadPrism 5 software.

#### Melting temperature determination

Circular Dichroism (CD) spectra were recorded using Jasco J-810 spectropolarimeter equipped with Pelletier type temperature control system (Jasco PTC-4235) in 1 mm thick quartz cell and using the Spectra Manager software. Measurements were carried out in 10 mM sodium phosphate buffer at pH 8 with a protein concentration of 0.1 mg/mL. For *wt Sis*Lac, denaturation was first recorded between 190 to 260 nm with a scattering speed of 20 nm/min every 10°C at temperatures ranging between 20 to 90°C. To determine the melting temperature of proteins (*wt Sis*Lac and variants), the denaturation was recorded at 222 nm by increasing the temperature from 20 to 90°C (at 5°C/min) in 10 mM sodium phosphate buffer at pH 8 containing increasing concentrations (0.5–3.5 M) of guanidinium chloride. The theoretical T_m_ without guanidinium chloride was extrapolated at the y-intercept by a linear fit using the GraphPadPrism 5 software.

### pH Dependence Profile Determination

The pH-dependence ethyl-paraoxonase activity (2 mM) profile of *Sis*Lac was monitored at 348 nm (the pH-independent isobestic point of *p*-nitrophenol and *p*-nitrophenoxide ion; ε_348nm_ = 5 300 M^−1^cm^−1^) [6]. To explore the pH range 5–11, different buffers were prepared containing 50 mM monobasic phosphate over the pH range 5–7, 50 mM HEPES for pH 8, 50 mM CHES over the pH range 9–10 and 50 mM dibasic phosphate at pH 11. The buffers also contained 150 mM NaCl and 0.2 mM of CoCl_2_, and were adjusted with NaOH or HCl. The kinetic measurements were performed at 25°C with a microplate reader (Tecan) and Magellan software in a 6.2 mm path length cell for 200 µL reaction in 96-well plate. Each experiment was made in triplicate and initial velocities were evaluated using Excel software (Microsoft).

### Crystallization and Structure Determination

The crystallization procedure of *Sis*Lac has been previously described [1]. Diffraction data were collected at the ESRF (Grenoble, France) BM-30A (FIP) beamline using a wavelength of 0.98 Å on an ADSC Quantum Q315 Detector. X-Ray diffraction data were integrated and scaled with the XDS program [36]. The presence of a twin was clearly established using *phenix.xtriage* [37] from the *PHENIX* refinement-program suite [38]. The Molecular replacement using the *Sso*Pox structure as model (PDB code 2vc5) was performed with *Phaser* [39]. The twin operator (*-h, -k, l*) and a twofold axis (z) arising from the twinning were determined using *phenix.xtriage*. The solution was then used for refinement performed using *REFMAC5* [40] using the twin option and *Coot* [41] for model improvement. The model and structure factor were deposited under the Protein Data Bank (PDB) code 4G2D. Despite the twinning, the electronic density maps were of good quality (**Fig. S3**; R and R_free_ values (0.2649 and 0.2925, respectively; **Table 1**)).

**Table 1:** Data collection and refinement statistics of *Sis*Lac structure.

Data collection	
PDB Id	4G2D
Wavelength (Å)	0.980
Detector	ADSC Q315
Oscillation (°)	0.5
Number of frames	323
Resolution (Å) (last bin)	2.70 (2.80-2.70)
Space group	P3_2_2_1_
Unit-cell parameters (Å)	a = 47.8, b = 47.8, c = 239.5α = 90.0, β = 90.0, γ = 120.0
No. of observed reflections (last bin)	86521 (8903)
No. of unique reflections (last bin)	9436 (959)
Completeness (%) (last bin)	99.9 (100)
R_merge_^a^ (%) (last bin)	6.2 (47.6)
R_meas_^b^ (%) (last bin)	6.6 (50.4)
I/σ(I) (last bin)	28.42 (4.71)
Redundancy (last bin)	9.17 (9.28)
Mosaicity (°)	0.103
**Refinement statistics**	
R_free_/R_work_ c	29.25/26.49
No. of total model atoms	2517
Ramachandran favored	98.2%
Ramachandran outliers	1.8%
Rmsd from ideal	
Bond lengths (Å)	0.0021
Bond angles (°)	0.5114

a
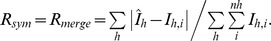

b
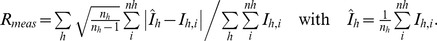

c *R_work_* = ∑||*F_0_*-|*F_c_*||/ = ∑|*F_0_*| where *F_0_* denotes the observed structure factor amplitude and *F_c_*, the structure factor amplitude calculated from the model. *R_free_* is as for *R_work_* but calculated with 5% of randomly chosen reflections omitted from the refinement.

Structural analysis and comparison, cartoon and ribbon representation were made using PyMOL (www.pymol.org). Surface contacts and interaction analysis was performed using the PROTORP server [42]. Root mean square deviations (r.m.s.d) were computed using Swiss-pdb-viewer software [43].

### Sequence Alignment

The alignment was performed using T-coffee server [44], [45], manually improved with *seaview* software [46] and finally drawn with *BioEdit* 7.1.3.

## Results

**Figure 2:**
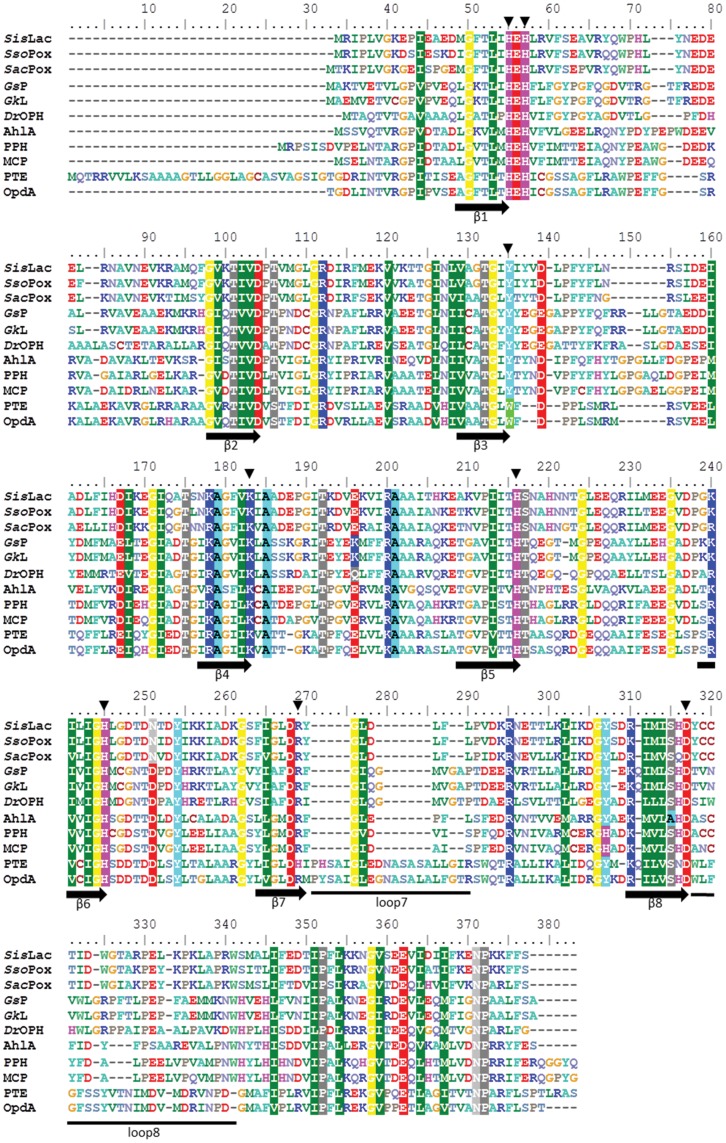
Sequence alignment of PLLs and PTEs. Sequence alignment of *Sis*Lac from *Sulfolobus islandicus* strain M.16.4 (ACR40964.1), *Sac*Pox from *Sulfolobus acidocaldarius* strain DSM 639 (AAY81433.1), *Sso*Pox from *Sulfolobus solfataricus* strain MT4 (AAW47234.1), *Gs*P from *Geobacillus stearothermophilus* strain 10, *Gk*L from *Geobacillus kaustophilus* strain HTA426 (YP_147359.1), *Dr*OPH from *Deinococcus radiodurans* (AAF10507.1), AhlA from *Rhodococcus erythropolis* (ACF57853.1), PPH from *Mycobacterium tuberculosis* (ACF57854.1), MCP from *Mycobacterium avium subsp. paratuberculosis* K-10 (NP_962602.1), *Pseudomonas diminuta* PTE and *Agrobacterium radiobacter* OpdA. Metal coordinating residues and important active site residues are indicated by a black vertical arrow. The β-sheets are indicated by a horizontal black arrow. An alignment of *Sulfolobal* PLL is provided in Supplementary information.

This study provides the characterization of *Sis*Lac isolated from *Sulfolobus islandicus* strain M.16.4. Several genomes of *S. islandicus* are available and encode highly similar (99% sequence identity) orthologs of *Sis*Lac (**Fig. S4**). Sequence comparison with close homologs *Sso*Pox (91% identical) from *Sulfolobus solfataricus* MT4 & P2 [6] and *Sac*Pox (76% identical) from *Sulfolobus acidocaldarius* DSM 639 [7] (**Fig. 2** **& S4**) reveals that the sequence divergence is mainly localized at the N-terminus and C-terminus of the protein. *Sis*Lac displays lower sequence identity with other members of the PLL family of proteins (35% *Gs*P/*Gk*L from Geobacillus sp. [8], [9], 28% *Dr*OPH form *Deinococcus radiodurans* [5], [34], 37–38% AhlA/PPH/MCP from mesophilic organisms [3], [4]) and ∼ 30% with PTEs (**Fig. 2**). Despite a very high sequence homology between *Sso*Pox and *Sis*Lac, both enzymes exhibit structural and enzymatic differences.

### Biochemical and Biophysical Characterization of SisLac

#### Oligomeric state analysis

**Figure 3:**
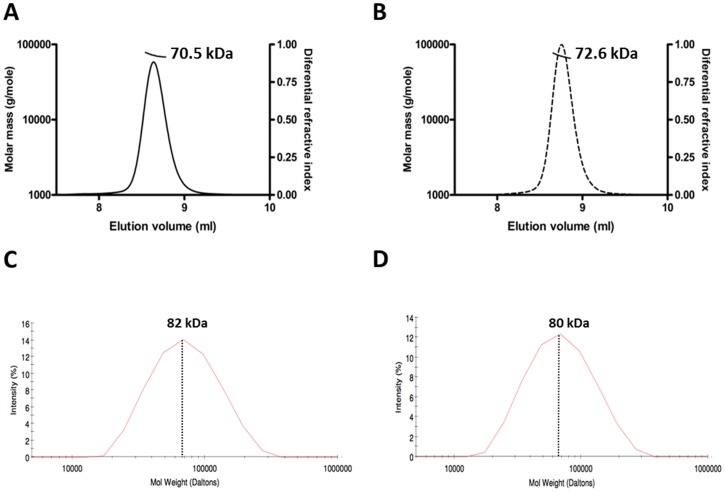
Oligomerization state analysis of *Sis*Lac. **A–B-** Multi-angle light scattering analysis of *Sso*Pox (**A.**) and *Sis*Lac (**B.**) at 25°C. The molecular weight of particules (dashed line) are represented in function of the elution profile (continuous line) during size exclusion chromatography. **C–D-** Dynamic light scattering experiments. Intensity (%) of the signal *versus* molecular weight of particles for *Sso*Pox sample (**C.**) and *Sis*Lac sample (**D.**).

Size exclusion chromatography, dynamic light scattering and multi angle light scattering experiments were carried out to determine the oligomeric states of *Sis*Lac and *Sso*Pox. Using a combination of static plus dynamic light scattering, UV spectrophotometry, and refractometry, *Sis*Lac and *Sso*Pox appear to be dimeric at room temperature (25°C) (**Fig. 3A–B**) (72.57±0.79 kDa and 70.46±0.97 kDa, respectively, *versus* MWs of *Sso*Pox and *Sis*Lac dimers = 71.2 kDa). Moreover, the dynamic light scattering experiments show apparent sizes for *Sis*Lac and *Sso*Pox of 80±3 kDa and 82±3 kDa, respectively (**Fig. 3C–D**). These results confirm that both proteins are dimeric at 25°C. The existence of homodimers is consistent with the crystal structures of *Sis*Lac, *Sso*Pox [47] and other PLLs (*Dr*OPH (PDB ID: 3FDK), *Gs*P (PDB ID: 3F4D) and *Gk*L (PDB ID: 3OJG)).

#### pH and temperature dependence

**Figure 4:**
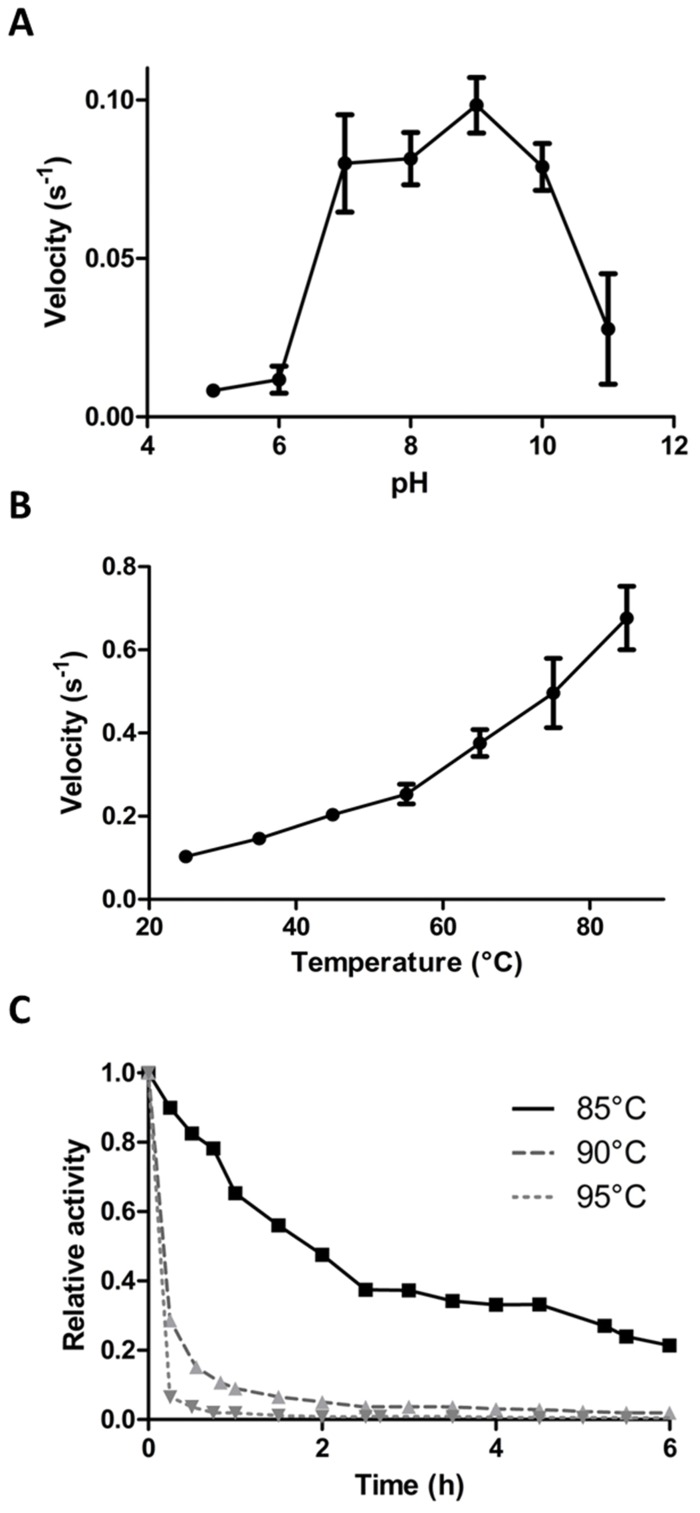
Biochemical analysis of *Sis*Lac. **A**- pH dependence of *Sis*Lac paraoxonase activity. Velocity obtained against 2 mM ethyl-paraoxon at different pH ranging between pH 5 and pH 11 (see *methods* for more details). **B**- Thermophilicity of *Sis*Lac. Initial rates for the paraoxonase activity of *Sis*Lac were measured at temperatures ranging from 25 up to 85°C. **C**- Thermal stability of *Sis*Lac. Relative residual activity of *Sis*Lac after different incubation at 85°C (*Black square*), 90°C (*grey triangle*) and 95°C (*Grey inverted triangle*) for different times yielded half-lives of 84 (±20) min for 85°C, 8.5 (±1.5) min for 90°C and 3.6 (±0.4) min for 95°C.

We determined the pH and temperature dependency of *Sis*Lac’s catalytic activities. However, because the lactonase assay utilizes a pH indicator, these characteristics could only be determined for the paraoxonase activity. The optimal pH for *Sis*Lac’s paraoxonase activity was established by measuring the velocity at pH ranging from 5 to 11 (**Fig. 4A**). The pH-rate dependence plot displays a bell-shape curve with a wide plateau between pH 7 and 10 (with maximal activity at pH 9). The same dependency what was described for *Sac*Pox at 70°C [7], and only a slightly different pattern was observed for *Sso*Pox (optimum at pH 8 [6]). The PTE from *P. diminuta* also exhibits an activity maximum at a pH range of 8–10 [48]. This observation is consistent with the hypothesis of common mechanism shared by these enzyme families.

The temperature dependency was investigated by measuring the paraoxonase activity at temperatures ranging from 25 to 85°C (**Fig. 4B**). The highest temperature tested (85°C) presented the highest velocity. However, within the tested temperature (imposed by technical limitations), we did not find a maximum. Similarly, no maxima were found in the cases of *Sso*Pox [6], *Dr*OPH [5] and *Gs*P [8]. In contrast, *Sac*Pox presents a maximum activity around 70°C and the activity decrease above this temperature [7].

### Thermostability

The thermostability of *Sis*Lac was evaluated based on its catalytic activity. The residual paraoxonase activity of the enzyme after different incubation times at 80, 90 and 95°C was measured (**Fig. 4C**). The enzyme exhibited respective half-lives of 84±20 min, 8.5±1.5 min and 3.6±0.4 min at 85, 90 and 95°C. In comparison, *Sso*Pox exhibits a half-life of 4 hours at 95°C, and 90 min at 100°C [6], and *Sac*Pox exhibits a half-life of 5 min at 90°C [7] (from *S. acidocaldarius,* living temperatures from 55 to 85°C [49]).

The thermal stability of *Sis*Lac was also determined by circular dichroism at temperatures ranging between 20°C and 90°C (**Fig. S5**). However, as previously observed for *Sso*Pox [47], the extreme thermostability of this enzyme does not allow to precisely determine a melting temperature (T_m_) by this method. Different concentrations of guanidium chloride were required to further destabilize the protein and the T_m_ values were extrapolated to zero guanidinium chloride: T_m_ at 102±2°C for *Sis*Lac (**Fig. S5**) (while *Sso*Pox’s T_m_ = 106°C [47]). These values are in the range of the other characterized thermostable PLLs, including *Dr*OPH and *Gs*P whose Tm values are 88.1°C [5] and 106.6°C [8], respectively. Notably, the extremophile *Deinoccocus radiodurans* is a mesophilic bacteria [5], and *Dr*OPH exhibits an extreme T_m_, typical for enzymes from hyperthermophiles or thermophiles (*Sulfolobus solfataricus* living temperatures from 50 to 87°C [49], *Sulfolobus islandicus* from 59 to 91°C [2], *Geobacillus stearothermophilus* from 37–70°C [50], [51]).

### Enzymatic Characterization of SisLac

#### Phosphotriesterase activity

Kinetic parameters were determined for paraoxon (**Fig. S1I**) at 70°C and 25°C (**Table 2**). The catalytic efficiency obtained at 70°C for *Sis*Lac (k_cat_/K_M_ = 6.98(±1.56)×10^2^ M^−1^s^−1^) is similar to that reported for *Sac*Pox [7] and *Sso*Pox [52] (**Table S3**). The catalytic efficiency at 25°C was about 2.5 folds lower than the efficiency at 70°C (2.60(±0.58)×10^2^ M^−1^s^−1^), and is higher than that of *Gs*P (5,47(±0.47)×10^1^ M^−1^s^−1^) [8], *Gk*L (4.5 M^−1^s^−1^; at 37°C) [9], MCP (4.1 M^−1^s^−1^) [4] and *Dr*OPH (1.39±0.11 M^−1^s^−1^) [5]. These promiscuous phosphotriesterase catalytic parameters of lactonases [3], [14] contrast with the diffusion limit-like second order rates of *P. diminuta* PTE with paraoxon as substrate (k_cat_/K_M_ ∼ 10^8^ M**^−^** ^1^s**^−^** ^1^) [11].

**Table 2:** Ethyl-paraoxonase activity of *Sis*Lac.

Conditions	*Sis*Lac		
	k_cat_ (s^−1^)	K_M_ (µM)	k_cat_/K_M_ (s^−1^M^−1^)
**25**°**C**	1.42±0.09	5439±873	2.60(±0.58)×10^2^
**25**°**C SDS 0.1%**	14.31±3.16	2005±728	7.14(±4.16)×10^3^
**25**°**C SDS 0.01%**	2.70±0.29	4248±999	6.36(±2.18)×10^2^
**70**°**C**	0.79±0.04	1131±196	6.98(±1.56)×10^2^

Data obtained with cobalt as cofactor.

The modulation of *Sis*Lac phosphotriester hydrolysis by Sodium Dodecyl Sulfate (SDS), which has been previously shown to act as an activator in the case of *Sso*Pox [53], was also tested (**Table 2**). Interestingly, the addition of 0.01% SDS yields to a 2.5 folds increase in the paraoxonase catalytic efficiency (6.36(±2.18)×10^2^ M^−1^s^−1^), whereas the addition of 0.1% SDS enhanced the efficiency by 25 folds (k_cat_/K_M_ = 7.14(±4.16)×10^3^ M^−1^s^−1^).

Others phosphotriesters (**Fig. 1A**) were also tested as substrates at 25°C; including methyl-paraoxon (**Fig. S1II**), parathion (**Fig. S1III**), methyl-parathion (**Fig. S1IV**), malathion (**Fig. S1V**) and CMP-coumarin (**Fig. S1VI**) (methylphosphonic acid 3-cyano-4-methyl-2-oxo-2H-coumarin-7-yl ester cyclohexyl ester (a cyclosarin derivative in which the fluoro substituent of cyclosarin has been replaced by a cyanocoumarin group [54])) (**Table 3** **& S4**). These assays showed that *Sis*Lac is over 10 times more efficient towards methyl-paraoxon than for (ethyl-)paraoxon (4.26(±1.74)×10^3^ M^−1^s^−1^ and 2.60(±0.58)×10^2^ M^−1^s^−^1, respectively). In a similar fashion, *Sis*Lac shows a clear preference for the methyl-parathion (3.57(±0.3)×10^1^ M^−1^s^−1^), as compared with ethyl-parathion for which no catalysis could be detected. This suggests that the bulkiness of the substituent groups of some phosphotriesters affects a catalytically efficient binding. This preference has also been observed for *Sso*Pox and *Sac*Pox at 70°C [7]. Moreover, *Sis*Lac exhibits a pronounced thiono-effect; methyl-paraoxon and methyl-parathion differ by only one atom (the oxon function on the phosphorus moiety in paraoxon is a thiono in parathion), *Sis*Lac hydrolyzes methyl-paraoxon about 100 times more efficiently (4.26(±1.74)×10^3^ M^−1^s^−1^ and 3.57(±0.3)×10^1^ M^−1^s^−1^, respectively). PTEs does not exhibit such a drastic difference and paraoxon is only a slightly better substrate than parathion [20], [55]. Kinetics parameters were also recorded for the hydrolysis of another sulfur-containing organophosphate, the insecticide called malathion (1.88±0.43 M^−1^s^−1^). *Sis*Lac exhibits a similar K_M_ for this substrate than for methyl-parathion, but malathion is a considerably slower substrate than parathion. Finally, *Sis*Lac hydrolyzes the nerve agent analog CMP-coumarin with moderate efficiency (4.26(±1.86)×10^3^ M^−1^s^−1^) illustrating its potential for organophosphorus detoxification.

**Table 3:** Phosphotriesterase activity of *Sis*Lac.

Substrate	k_cat_ (s^−1^)	K_M_ (µM)	k_cat_/K_M_ (s^−1^M^−1^)
**Ethyl-Paraoxon (I)**	1.42±0.09	5 439±873	2.60(±0.58)×10^2^
**Methyl-Paraoxon (II)**	7.40±1.26	1 739±417	4.26(±1.74)×10^3^
**Ethyl-Parathion (III)**	ND	ND	ND
**Methyl-Parathion (IV)**	9.7(±0.2)×10^−3^	272±17	3.57(±0.30)×10^1^
**Malathion (** ***r*** **) (V)**	6.2(±0.4)×10^−4^	330±54	1.88±0.43
**CMP (** ***r*** **) (VI)**	1.86±0.27	437±130	4.26(±1.86)×10^3^

*r* corresponds to racemic solution. Data obtained with cobalt as cofactor. ND corresponds to substrates for which no hydrolysis can be detected. Roman numeration corresponds to chemical structures of **Fig. S1**.

#### Esterase activity

Although lactones constitute a specific class of esters (**Fig. 1B**), no esterase activity was detected within PLL family members with the exception of *Sso*Pox and *Sac*Pox (with 2-naphthyl acetate [6], [7], *p*-nitrophenyl butanoate and 2-naphthyl acetate [3]). It is therefore not surprising that *Sis*Lac hydrolyzes *p*-nitrophenyl-acetate (**Fig. S1VIII**) with k_cat_/K_M_ = 1.6(±0.5)×10^3^ M^−1^s^−1^, a 50 times higher catalytic efficiency than *Sso*Pox (k_cat_/K_M_ of 3.12(±0.33)×10^1^ M^−1^s^−1^) (**Table 4**). However, both proteins do not exhibit any detectable activity against phenyl-acetate (**Fig. S1VII**), *p*-nitrophenyl-decanoate (**Fig. S1IX**), nitrophenyl-acetate (**Fig. S1X**) and 4-acetoxyacetophenone (**Fig. S1XI**) (**Table 4**).

**Table 4:** Esterase activity of *Sis*Lac.

Substrat	*Sis*Lac			*Sso*Pox		
	k_cat_ (s^−1^)	K_M_ (µM)	k_cat_/K_M_ (s^−1^M^−1^)	k_cat_ (s^−1^)	K_M_ (µM)	k_cat_/K_M_ (s^−1^M^−1^)
**Phenyl-acetate (VII)**	ND	ND	ND	ND	ND	ND
***p*** **NP-acetate(VIII)**	0.20±0.01	124±36	1.6(±0.5)×10^3^	0.17±0.007	5447±352	3.12(±0.33)×10^1^
***p*** **NP-decanoate (XI)**	ND	ND	ND			
***m*** **NP-acetate (X)**	ND	ND	ND	ND	ND	ND
**4AAP (XI)**	ND	ND	ND	ND	ND	ND

*p*NP corresponds to *para*-nitrophenol leaving group and *m*NP to *meta*-nitrophenol leaving group. Data obtained with cobalt as cofactor. ND corresponds to substrates for which no hydrolysis can be detected. Roman numeration corresponds to chemical structures of **Fig. S1**.

#### Lactonase activity

PLLs are lactonases that might be involved in *quorum* quenching mechanisms [3], [13]. We thus assayed the activity of *Sis*Lac on several AHLs (**Fig. 1C–D**) of different chain lengths with the aim of evaluating *Sis*Lac’s specificity (**Table 5**). These experiments revealed that *Sis*Lac exhibits clear preference for AHLs with medium-length aliphatic chains (C8 and C10-AHL). Long chains are strongly disfavored, the efficiency of 3-oxo-C12-AHL hydrolysis (**Fig. S2VI**) (8.97(±3.45)×10^2^ M^−1^s^−1^) is 100 fold lower than that of 3-oxo-C10-AHL (**Fig. S2V**) (9.63(±1.89)×10^4^ M^−1^s^−1^). In addition, 3-oxo-AHLs (**Fig. 1D**) are overall better substrates for *Sis*Lac than unsubstituted (**Fig. 1C**).

**Table 5:** AHL lactonase activity of *Sis*Lac.

Substrate	*Sis*Lac		
	k_cat_ (s^−1^)	K_M_ (µM)	k_cat_/K_M_ (s^−1^M^−1^)
**C4-AHL (** ***r*** **) (I)**	ND	ND	3.89±0.60
**C8-AHL (** ***r*** **) (II)**	0.71±0.09	677±163	1.05(±0.39)×10^3^
**C12-AHL (** ***r*** **) (III)**	1.90±0.79	1 303±1 078	1.46(±1.81)×10^3^
**3-oxo-C8-AHL (** ***r*** **) (IV)**	4.10±0.09	42±7	9.70(±1.84)×10^4^
**3-oxo-C10-AHL (** ***l*** **) (V)**	10.65±0.36	111±18	9.63(±1.89)×10^4^
**3-oxo-C12-AHL (** ***l*** **) (VI)**	0.39±0.04	435±123	8.97(±3.45)×10^2^

*r* corresponds to racemic solution and *l* at the pure levorotatory enantiomer. Data obtained with cobalt as cofactor. ND corresponds to not determined value. Roman numeration corresponds to chemical structures of **Fig. S2**.

Others lactones were also assayed as substrates (**Table 6**), such as the γ-lactones (5 atoms lactone ring) (**Fig. 1E**), δ-lactones (6-atoms lactone ring) (**Fig. 1F**) and ε-lactone (7 atoms lactone ring) (**Fig. S2XI**), with alkyl substituent on carbons 4 and 5 of the lactone ring (contrary to the substitution of carbon 2 in AHLs (**Fig. 1C**) (**Table 6**). Finally, dihydrocoumarin (**Fig. S2XII**), an aromatic lactone, was also tested (**Table 6**). We found that γ-lactones (**Fig. 1E**) and δ-lactones (**Fig. 1F**) comprise good substrates for *Sis*Lac, δ-lactones being the preferred substrates. Indeed, the best δ-lactone (undecanoic-δ-lactone (**Fig. S2XIV**), 1.77(±0.04)×10^6^ M^−1^s^−1^) is hydrolyzed with over 5-times higher catalytic efficiency than the best γ-lactone (nonanoic-γ-lactone (**Fig. S2IX**), 2.04(±1.12)×10^5^ M^−1^s^−1^). The latter is 2 times a better substrate than the best AHL substrate (3-oxo-C8-AHL (**Fig. S2IV**), 9.70(±1.84)×10^4^ M^−1^s^−1^). Comparison of different lactones possessing different alkyl chain lengths confirmed the trend observed for AHLs, whereby acyl chains containing 7 carbons were preferred by the enzyme. For γ-lactones and δ-lactones, the alkyl chain length preferred by the enzyme is between 5 and 6 carbon atoms, and a similar specificity was observed for MCP, *Gk*L and *Dr*OPH enzymes [4], [9], [34]. Interestingly, whereas the short chain C4-AHL (**Fig. S2I**) is a poor substrate for *Sis*Lac, the γ-heptanolide lactone (**Fig. S2VIII**) (3 carbon atoms in the alkyl side-chain) shows a 10^4^ higher catalytic efficiency. In fact, lactones with very short or without side-chains (dihydrocoumarin (**Fig. S2XVII**), γ-butyrolactone (**Fig. S2VII**), δ-valerolactone (**Fig. S2XII**), ε-caprolactone (**Fig. S2XVI**)) constitute better substrates than C4-AHL (**Fig. S2I**).

**Table 6:** Oxo-lactone lactonase activity of *Sis*Lac.

Family	Substrate	*Sis*Lac		
		k_cat_ (s^−1^)	K_M_ (µM)	k_cat_/K_M_ (s^−1^M^−1^)
**γ-lactone**	**γ-butyrolactone (VII)**	5.75±0.63	158±38	3.64(±1.27)×10^4^
	**γ-heptanolide (** ***r*** **) (VIII)**	5.89±0.07	128±10	4.61(±0.09)×10^4^
	**Nonanoic-γ-lactone (** ***r*** **) (IX)**	3.10±0.13	15±8	2.04(±1.12)×10^5^
	**Undecanoic-γ-lactone (** ***r*** **) (X)**	2.15±0.13	391±90	5.49(±1.59)×10^3^
	**Dodecanoic-γ-lactone (** ***r*** **) (XI)**	1.49±0.08	475±83	3.14(±0.72)×10^3^
**δ-lactone**	**δ-valerolactone (XII)**	0.43±0.09	1 949±1 056	2.20(±1.65)×10^2^
	**Nonanoic-δ-lactone (** ***r*** **) (XIII)**	51.70±1.67	62±22	8.28(±3.14)×10^5^
	**Undecanoic-δ-lactone (** ***r*** **) (XIV)**	17.65±0.38	<10	>1.77(±0.04)×10^6^
	**Dodecanoic-δ-lactone (** ***r*** **) (XV)**	11.09±0.71	124±36	8.95(±3.16)×10^4^
**Others**	**ε-caprolactone (XVI)**	7.27±0.31	367±54	1.98(±0.38)×10^4^
	**Dihydrocoumarin (XVII)**	11.50±0.37	1 122±75	1.04(±1.02)×10^4^

Data obtained with cobalt as cofactor. Roman numeration corresponds to chemical structures of **Fig. S2**.

### Structural Analysis of SisLac

*Sis*Lac is homodimeric in the crystal structure with overall dimensions of the monomers of being approximately 39×48×56 Å. As for its close homolog *Sso*Pox [13] and related PLLs like *Dr*OPH [5], *Gs*P [8] and *Gk*L [9], *Sis*Lac is roughly globular and exhibits a (β/α)_8_ barrel topology. The active site consists of a binuclear center located at the C-Terminal of the barrel. Four histidines (His22, His24, His170, His199), one aspartic acid (Asp256) and a carboxylated lysine (residue 137) are coordinating the two metals. The two metal cations (possibly iron and cobalt, as seen for *Sso*Pox [13] and OpdA [20]) are bridged by a water molecule that is presumed to be the catalytic nucleophile. The active site includes a long hydrophobic channel that was revealed by structural studies on *Sso*Pox as the binding region of aliphatic chains for the AHL substrates [13]. Indeed, *Sis*Lac’s structure is overall very similar to the structure of *Sso*Pox (root-mean-square deviation (r.m.s.d.) for α-carbon atoms (over 314 atoms) of 0.35 Å).

#### Salt bridge network analysis

**Figure 5:**
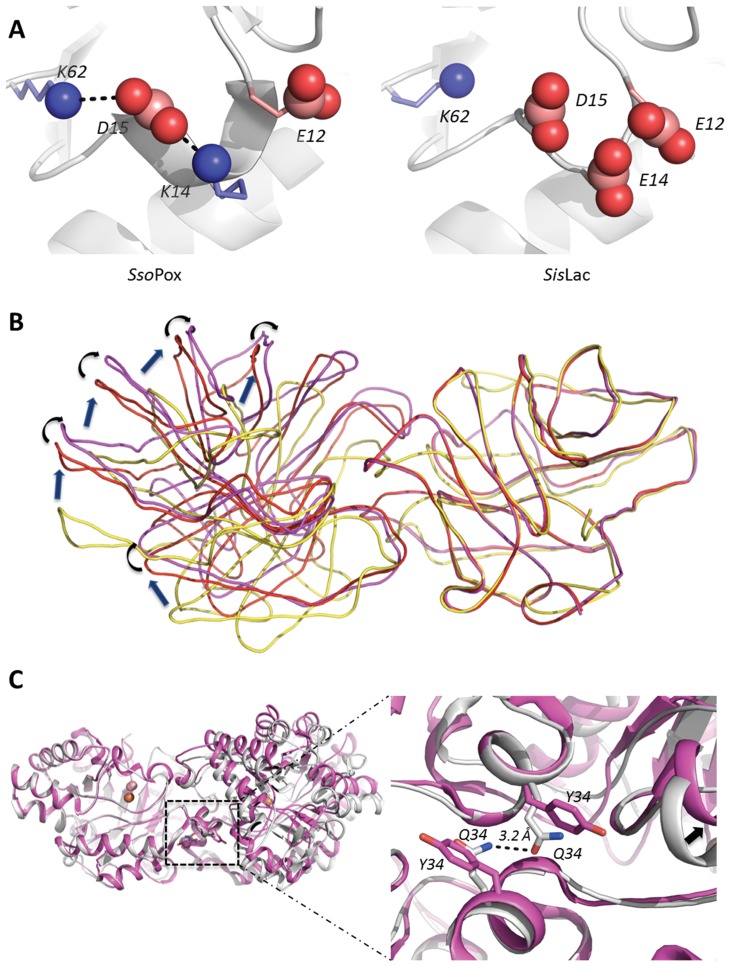
Structural analysis. **A**- Close view on position 14 in *Sso*Pox (left side) and *Sis*Lac (right side) structures. Negatively and positively charged residues are represented as red and blue spheres, respectively. Black dashed lines correspond to the putative salt-bridges in *Sso*Pox. **B**- Structural comparisons of the dimers of *Sis*Lac (*violet*), *Sso*Pox (*red*) and *P. diminuta* PTE (*yellow*). One monomer was used to superpose the three structures (shown on the right side), thus indicating the conformational shift in the position of the second monomer (left side). The differences are highlighted by blue arrows (*P. diminuta* PTE *versus Sso*Pox) or by black arrows (*Sso*Pox *versus Sis*Lac). When compared to *P. diminuta* PTE, the rotational shift observed in *Sis*Lac is more pronounced than that of *Sso*Pox. **C**- Close view on position 34 in *Sso*Pox (grey) and *Sis*Lac (violet). Black dashed lines correspond to the hydrogen bond between Q34 of each monomer of *Sso*Pox with a distance of 3.2 Å. The black arrow shows the rotational shift that seems to be induced by this substitution.

*Sis*Lac sequence exhibits approximately the same amino acid content as *Sso*Pox, containing 14.3 *versus* 16% of uncharged polar residues and 28.7 *versus* 28% of charged residues. This is no surprising since both enzymes possess high sequence identity (91%) (see sequence alignment, **Fig. 2**). As described for *Sso*Pox, the charged residues are mainly located at the protein surface, forming complex electrostatic networks [47] that includes 28 salt bridges implicating 46 residues. This charge network mainly differs by the substitution K14E in *Sis*Lac that suppress a salt bridge network between E12-K14-D15 of *Sso*Pox, and consequently creates a local concentration of 3 negative charges within 4 consecutive residues (**Fig. 5A**).

#### Dimer interface analysis

The dimer interface of *Sis*Lac comprises 46 residues (44 in *Sso*Pox). The contacting area is almost identical and comprise a typical value for homodimers [42] (1770 Å^2^ for *Sis*Lac structure, 1750 Å^2^ for *Sso*Pox structure, lower for other thermostable PLLs: 1728 Å^2^ for *Gk*L structure, 1632 Å^2^ for *Gs*P structure and 1473 Å^2^ for *Dr*OPH structure). The nature of this interface is mainly hydrophobic in both enzymes, but *Sis*Lac’s interface tends to be more hydrophobic (56% for *Sis*Lac, 52.6% for *Sso*Pox), more charged (*Sis*Lac 23.91%, *Sso*Pox 20.45%) and less polar (*Sis*Lac 32.61%, *Sso*Pox 36.36%). Four interface residues differ from *Sis*Lac to *Sso*Pox but these substitutions do not fully explain the observed differences. Interestingly, the superposition of *Sis*Lac and *Sso*Pox monomers shows a relative movement of the second *Sis*Lac monomer of about 5 Å relative to *Sso*Pox’s, revealing that the interface area is slightly shifted (**Fig. 5B**). This reorganization of the dimer interface appears to be due to the substitution Q34Y, although it could also originate from the different crystal packing of both proteins. Whereas the two Q34 interact with each other’s in *Sso*Pox structure, the bulkiness of both Y34 in *Sis*Lac imposes a reorientation of the dimer (**Fig. 5C**). This reorientation increases the monomers interpenetration, and makes one closer to the active site of the other. This trend was previously described while comparing the *P. diminuta* PTE and *Sso*Pox structures [47], although these enzymes are far more divergent in sequence (about 30% identity). Although the biological importance and catalytic influence of dimerization for these enzymes remain unclear, second shell active site residues are notably involved in dimer formation.

### Key Substitutions between SisLac and SsoPox

The structural analysis revealed that the positions 14 and 34 seem to be the major impacting variations between *Sis*Lac and *Sso*Pox structures, that may relate to dimerization changes (position 34) or stability (positions 14, 34). Mutational intermediates between *Sis*Lac and *Sso*Pox have been constructed to evaluate the consequences of these substitutions (E14K and Y34Q) for the enzyme stability and activity. All the variants exhibit lower Tm as compared to *Sis*Lac (102±2°C) and *Sso*Pox (106°C) [6] while double variants (Y34Q-E14K) present the highest Tm among the variants (**Table 7**). The analysis of Tm reveals that the substitution Y34Q and E14K are destabilizing on *Sis*Lac background but the combination of both variations tends to restore partially the stability (**Table 7**). Additionally, the mutants have been characterized for catalytic activity against ethyl/methyl-paraoxon and for the best AHL, δ-lactone and γ-lactone substrates of *wt Sis*Lac (**Table 8**). The efficiency of methyl-paraoxon hydrolysis is similar for *wt* and the mutants, whereas the mutants exhibit higher catalytic efficiency against ethyl-paraoxon than the *wt* enzyme. However, the mutants exhibit a dramatically reduced AHLase activity (**Table 8**). A similar trend is observed with δ/γ-lactones. These results clearly highlight the critical importance of these positions for *Sis*Lac stability and activity, and validate our structural analysis.

**Table 7:** Melting temperature of *Sis*Lac and its variants.

Protein	Tm (°C)
*Sis*Lac *wt*	102±2
*Sis*Lac E14K	96±1
*Sis*Lac Y34Q	94±2
*Sis*Lac E14K-Y34Q	98±2

**Table 8:** Kinetic characterization of mutational intermediates between *Sis*Lac and *Sso*Pox.

Substrate	Protein	k_cat_ (s^−1^)	K_M_ (µM)	k_cat_/K_M_ (M^−1^s^−1^)
**Ethyl Paraoxon**	*Sis*Lac *wt*	1.42±0.09	5439±873	2.60(±0.58)×10^2^
	*Sis*Lac E14K	0.14±0.01	30±5	4.50(±0.80)×10^3^
	*Sis*Lac Y34Q	0.13±0.01	150±12	8.66(±0.89)×10^2^
	*Sis*Lac E14K-Y34Q	0.17±0.01	132±19	1.28(±0.22)×10^3^
**Methyl Paraoxon**	*Sis*Lac *wt*	7.40±1.26	1739±417	4.26(±1.74)×10^3^
	*Sis*Lac E14K	0.30±0.01	50±6	6.00(±0.80)×10^3^
	*Sis*Lac Y34Q	0.42±0.01	261±24	1.61(±0.19)×10^3^
	*Sis*Lac E14K-Y34Q*	0.86±0.21	620±198	1.39(±0.78)×10^3^
**3-oxo-C8-AHL (** ***r*** **)**	*Sis*Lac *wt*	4.1±0.09	42±7	9.70(±1.84)×10^4^
	*Sis*Lac E14K	0.85±0.06	917±150	9.27(±2.17)×10^2^
	*Sis*Lac Y34Q	0.92±0.05	1214±135	7.58(±1.25)×10^2^
	*Sis*Lac E14K-Y34Q	0.97±0.04	1017±98	9.54(±1.31)×10^2^
**nonanoic-γ- lactone (** ***r*** **)**	*Sis*Lac *wt*	3.10±0.13	15±8	2.04(±1.12)×10^5^
	*Sis*Lac E14K	1.88±0.07	25±10	7.52(±3.29)×10^4^
	*Sis*Lac Y34Q	1.91±0.05	61±9	3.13(±0.17)×10^4^
	*Sis*Lac E14K-Y34Q	1.99±0.12	47±18	4.23(±1.87)×10^4^
**Undecanoic-δ- lactone (** ***r*** **)**	*Sis*Lac *wt*	17.65±0.38	<10	>1.77(±0.04)×10^6^
	*Sis*Lac E14K	14.09±0.59	42±16	3.35(±1.41)×10^5^
	*Sis*Lac Y34Q	12.80±0.51	121±22	1.06(±0.23)×10^5^
	*Sis*Lac E14K-Y34Q	12.91±0.58	43±13	3.00(±1.04)×10^5^

Data obtained with cobalt as cofactor. * Hydrolysis of methyl-paraoxon by *Sis*Lac E14K-Y34Q exhibits a substrate inhibition profile with K_I_ = 855±376 µM.

## Discussion

### Catalytic Properties of *Sis*Lac

The pH dependence of *Sis*Lac was investigated and yields a bell-shaped curve with a pH optimum at pH 9, a consistent behavior with previously characterized PLLs [6], [7] and PTEs [48]. This pH dependence profile is also in agreement with the commonly accepted hydrolysis mechanism where a water molecule activated by the bi-metallic active site serves as nucleophile [13]. Additionally, the metal dependence was assayed and the metal nature was found to modulate the catalytic activities, as previously described in PTEs [19] and PLLs [34]. Amongst the tested metals, *Sis*Lac shows preference for cobalt cations for both lactonase and paraoxonase activities (**supplementary information & Fig. S6**), as previously reported for the paraoxonase activity of *Sso*Pox [6] and the lactonase activity of *Dr*OPH [34], MCP [4] and *Gk*L [9]. The metal dependence of *Sis*Lac may be related to the relative pK_a_ of considered metal with H_2_O, since the pK_a_ of Co^2+^/H_2_O is lower than that of Zn^2+^/H_2_O and Mn^2+^/H_2_O (8.9 *versus* 9.0, and 10.6, respectively [20]), thus Co^2+^ would better contribute to the activation of the nucleophile. In addition, Co^2+^ is more electronegative than Zn^2+^ and Mn^2+^ (1.88 *versus* 1.65 and 1.55, respectively [19]), thus being more efficient for stabilizing the developing negative charge on the transition state.

### Phosphotriesterase Activity

The catalytic efficiency of paraoxon hydrolysis by *Sis*Lac at 25°C (k_cat_/k_M_ = 2.60(±0.58)×10^2^ M^−1^s^−1^) shows that *Sis*Lac is endowed with one of the highest paraoxonase activity amongst PLLs [3], [4], [5], [6], [8], [9]. Moreover, this activity can be considerably increased by addition of 0.1% SDS (25 folds), suggesting that the enzyme has an interesting potential for catalytic improvement. The potential of this enzyme for organophosphorus compounds bio-decontamination is further illustrated by its ability to hydrolyze the nerve agent analog CMP-coumarin with significant efficiency (k_cat_/K_M_ = 4.26(±1.86)×10^3^ M^−1^s^−1^).

Interestingly, we observed that methyl-paraoxon is a better substrate than ethyl-paraoxon, the same trend being monitored between methyl and ethyl-parathion. The fact that smaller substituents on the phosphorus center are preferred by the enzyme is consistent with the promiscuous nature of paraoxonase activity in *Sis*Lac. Moreover, the higher compaction of the *Sis*Lac monomer makes active sites closer one to each other in the dimer as compared to *Sso*Pox structure and thus could explain the substrate preference in disfavor of bulkier substrates.

In addition, *Sis*Lac exhibits very low catalytic efficiency towards P = S containing organophosphates (*e.g.* ethyl and methyl-parathion, malathion). The K_M_ values are in the range of the native substrates (hundreds of µM), but the k_cat_ values are extremely low (about 3 orders of magnitude lower than P = O containing OPs), which is a typical behavior for promiscuous activities [56]. PTEs, albeit preferring paraoxon to parathion as a substrate, do not exhibit such a pronounced thiono-effect [20], [55]. PTEs constitutes a protein family that are believed to have diverged from PLLs like *Sis*Lac [3] in the last few decades to specifically hydrolyzes man-made insecticides [57]. They thus may have evolved to suppress this thiono-effect in order to hydrolyze the most used pesticides (e.g. parathion).

### Esterase and Lactonase Activity

PLLs have been previously characterized as poor esterases [3], and so is *Sis*Lac. Amongst the five tested esters, only *p*NP-acetate is a substrate for *Sis*Lac. The natural substrates of PLLs, lactones, being a specific class of esters, it is thus surprising that PLLs exhibit low esterase activity. It might results from a rather good binding to the active site (as suggested by observed K_M_ for *p*NP-acetate), but with a large fraction of non-productive binding (very low k_cat_).

PLLs are natural lactonases that might be involved in *quorum* quenching [13]. Their precise substrates and biological function(s) are however still unknown. Lactones encompass two major families of compounds, the lipophilic aroma (oxo-lactones) and the Acyl Homoserine Lactones (AHLs) involved in *quorum* sensing. The *quorum* sensing is common in bacteria, but its existence in the archaeal world remains unclear, despite the finding of AHL-based *quorum* sensing stimulating molecules in *Natronoccocus occultus* [58], the presence of biofilms in *Sulfolobus sp.* [59] and the recent characterization of complete carboxylated-AHLs *quorum* sensing system in methanogenic archaeon [60].

Our kinetic experiments show that *Sis*Lac prefers long aliphatic chain lactones, exhibiting optimal activity when the acyl chain contains 7 carbon atoms, as seen for *Sso*Pox [52]. *Sis*Lac show also preference for 3-oxo-AHLs and hydrolyzes poorly short chain AHLs. It is interesting to notice that other PLLs, like AhlA and PPH, hydrolyze short and long chain lactones with similar catalytic efficiency [3]. It is thus possible that within the PLL family, different sub-groups of enzymes exhibit different specificities, and thus possibly different physiological functions. In addition, we show that *Sis*Lac is a proficient enzyme against oxo-lactones (best substrate: undecanoic-δ-lactone, k_cat_/k_M_ = 1.77(±0.04)×10^6^ M^−1^s^−1^), and hydrolyzes more efficiently long chain lactones, with a preference for 5 to 6 acyl chain carbon atoms. This preference is similar to that observed for AHLs as substrates, and possibly indicates that oxo-lactones and AHLs acyl chains bind into a similar pocket, most likely the hydrophobic channel connected to the active site that was depicted for *Sso*Pox structure [13], [47]. However, interestingly, whereas the short chain C4-AHL is a poor substrate for *Sis*Lac, the heptanolide-δ-lactone (3 carbon atoms in the acyl chain) shows a 10^4^ higher hydrolysis efficiency. In addition, lactones with very short or without acyl chains (dihydrocoumarin, γ-butyrolactone, δ-valerolactone, ε-caprolactone) constitute better substrates than C4-AHL. Altogether, these features might reveal that γ- and δ-lactones utilize an alternate binding mode for the lactone ring than AHLs, and/or that C4-AHL does not bind in a catalytically relevant fashion to *Sis*Lac.

### Structural Determinants for Thermal Stability

The major structural determinants explaining the high thermal stability of hPLLs have been documented with the example of *Sso*Pox [47], the comparison with *Gs*P and *Dr*OPH enzymes [8], and are part of the classical properties described for hyperthermostable proteins [61]. The structures of these enzymes, including *Sis*Lac, exhibit a high number of salt bridges organized in complex networks of charges at the protein surface that may rigidify the global protein architecture. Moreover, the homodimer interface is larger and more hydrophobic (see Results) and the overall structure is more compact than mesophilic counterparts [47]. Here we observed that the interface area between *Sis*Lac’s monomers is slightly shifted, as compared with *Sso*Pox structure. This reorganization of the dimer interface is consistent with observations made in solution. Indeed, as observed in crystals, both enzymes are dimeric at 25°C. The importance of hydrophobic contacts within the dimer interface of *Sis*Lac and *Sso*Pox explain the dimerization of the proteins. Since the hydrophobic effect increases with temperature, it is highly probable that these enzymes could be dimers at physiological temperatures (50–90°C) [2], [49].

Interestingly, both *Sulfolobus* species from which *Sis*Lac and *Sso*Pox enzymes originates lives in similarly extreme environments (*S. solfataricus* from 50 to 87°C [49], *S. islandicus* from 59 to 91°C [2]) and exhibit similar thermostability (T_m_ = 102±2°C for *Sis*Lac and T_m_ = 106°C for *Sso*Pox [6]). Taking advantage from their high sequence identity between the two proteins, we studied the substitution K14E in *Sis*Lac (as compared to *Sso*Pox) that breaks a salt bridge network at the C-terminus of the protein, a region concentrating the highest divergences among hPLLs as revealed by sequence alignment (*i.e. Sso*Pox, *Sac*Pox and *Sis*Lac). E14 engenders in *Sis*Lac a cluster of 3 negatively charged residues at the surface of the structure allowing to evaluate the contribution of these electrostatic interactions to the enzyme stability and activity. Moreover, another key substitution occurred between the two enzymes in the homodimerization interface. Q34Y is indeed a key substitution in the interface since it consists in a “pivot” residue, *i.e*. a residue that contacts its equivalent in the second protein molecule while forming the dimer, and seems to be responsible for the observed dimerization shift between *Sis*Lac and *Sso*Pox structures. Q34Y is, moreover, the substitution between *Sis*Lac and *Sso*Pox that is the closest in space to the active site (second shell). We therefore studied the effects of this variation on *Sis*Lac’s activity and stability.

Surprisingly, whereas K14 in *Sso*Pox is involved in a large network of charged interactions and may contribute to the overall protein rigidity [47], the variation E14K in *Sis*Lac is destabilizing (Tm is decreased by 6°C). The variation of the pivot interface residue Y34Q is also destabilizing on *Sis*Lac background (decrease of Tm by 8°C). Interestingly, the double variant E14K-Y34Q that carries two destabilizing mutations exhibits a higher Tm than the single variants, revealing the highly epistatic nature of these positions. Moreover, whereas the promiscuous phosphotriesterase activity is not altered by these substitutions, the lactonase activity, especially the AHLase activity is considerably reduced (by ∼100 folds) as compared to *wt*. The influence of E14K on *Sis*Lac’s catalytic activity is not obvious from a structural analysis. However, position 34 comprises a second shell residue, and the overall dimer interface is in the vicinity of the active site. Mutation of position 34 highly influences the protein dimerization and thus the degree of monomers interpenetration. Monomer active sites being close one to each other, their interpenetration could influence substrate specificities and catalytic efficiencies by fine steric or dynamic constraints which can’t be evaluated by structural analysis. These identified key substitutions, however, does not fully explain the observed different catalytic properties of *Sis*Lac and *Sso*Pox. The active site residues and configuration of these two enzymes being similar, these discrepancies might be partly mediated by yet unidentified substitutions distant from the active site.

Reconstructed mutational intermediates between *Sis*Lac and *Sso*Pox have lower fitness both in term of stability and AHLase activity. Despite the very high sequence identity between both proteins (91%), it may indicate that the evolutionary route that links them already comprise a fraction of highly epistatic mutations. In other words, the mutations that accumulate at a very early stage of divergence might not only be neutral but a fraction of them are highly cooperative.

## Supporting Information

Figure S1 **Chemical structure of phosphotriesters (I-VI) and esters (VII-XI).**
(DOC)

Figure S2 **Chemical structure of AHLs (I-VI), γ-lactones (VII-XI), δ-lactones (XII-XV) and other lactones (XVI-XVII).**
(DOC)

Figure S3 **Electronic density map of** ***Sis*** **Lac at 2.7 Å resolution.**
(DOC)

Figure S4 **Sequence alignment of PLLs from Sulfolobus species.**
(DOC)

Figure S5 **Thermostability analysis of** ***Sis*** **Lac by circular dichroism.**
(DOC)

Figure S6 ***Sis*** **Lac metal preference.**
(DOC)

Information S1 **Supplementary information for** ***Sis*** **Lac metal preferences.**
(DOC)

Table S1 **Primers used for site directed mutagenesis.**
(DOC)

Table S2 **Kinetics protocols.**
(DOC)

Table S3 **Ethyl-paraoxonase comparison between** ***Sso*** **Pox,** ***Sac*** **Pox and** ***Sis*** **Lac.**
(DOC)

Table S4 **Phosphotriesterase activity comparison between** ***Gs*** **P,** ***Dr*** **OPH and** ***Sso*** **Pox and** ***Sis*** **Lac.**
(DOC)
