# Supplementary material for: Structural and Enzymatic characterization of the lactonase SisLac from Sulfolobus islandicus
Source: PLoS One. 2012 Oct 10;7(10):e47028. doi: 10.1371/journal.pone.0047028 (PMC3468530; doi:10.1371/journal.pone.0047028)
Supplement: Figure S1 — Chemical structure of phosphotriesters (I-VI) and esters (VII-XI). (DOC) [file pone.0047028.s001.doc]

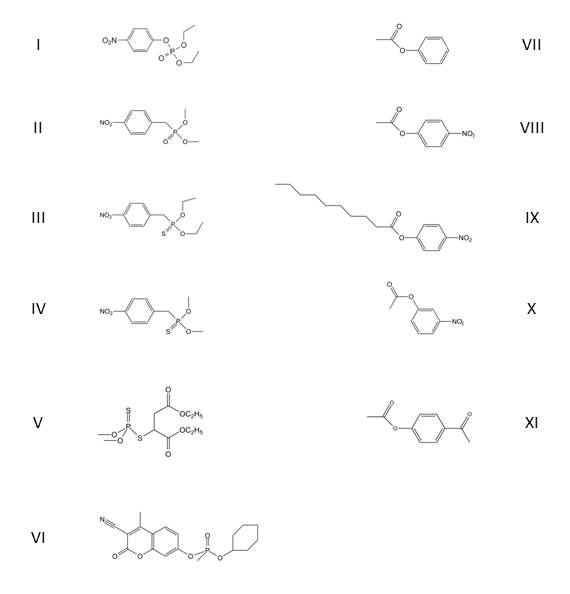


**Figure S1: Chemical structure of organophosphorous compounds (I-VI) and esters (VII-XI)**

Chemical structure of ethyl-paraoxon (I), methyl-paraoxon (II), ethyl-parathion (III), methyl-parathion (IV), malathion (V), CMP (VI), phenyl-acetate (VII), *p*NP-acetate (VIII), *p*NP-decanoate (IX), *m*NP-acetate (X) and 4-acetoxy-acetophenone (4AAP; XI).
