# Supplementary material for: Structural and Enzymatic characterization of the lactonase SisLac from Sulfolobus islandicus
Source: PLoS One. 2012 Oct 10;7(10):e47028. doi: 10.1371/journal.pone.0047028 (PMC3468530; doi:10.1371/journal.pone.0047028)
Supplement: Figure S2 — Chemical structure of AHLs (I-VI), γ-lactones (VII-XI), δ-lactones (XII-XV) and other lactones (XVI-XVII). (DOC) [file pone.0047028.s002.doc]

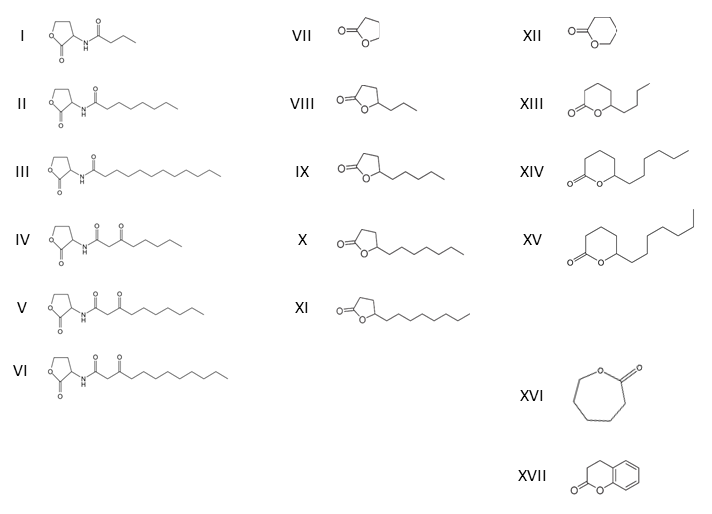


**Figure S2: Chemical structure of AHLs (I-VI), γ-lactones (VII-XI), δ-lactones (XII-XV) and other lactones (XVI-XVII)**

Chemical structure of C4-AHL (I), C8-AHL (II), C12-AHL (III), 3-oxo-C8 AHL (IV), 3-oxo-C10 AHL (V), 3-oxo-C12 AHL (VI), γ-butyrolactone (VII), γ-heptanolide (VIII), Nonanoic-γ-lactone (IX), Undecanoic-γ-lactone (X), Dodecanoic-γ-lactone (XI), δ-valerolactone (XII), Nonanoic-δ-lactone (XIII), Undecanoic-δ-lactone (XIV), Dodecanoic-δ-lactone (XV), ε-caprolactone (XVI) and Dihydrocoumarin (XVII).
