# Supplementary material for: Structural and Enzymatic characterization of the lactonase SisLac from Sulfolobus islandicus
Source: PLoS One. 2012 Oct 10;7(10):e47028. doi: 10.1371/journal.pone.0047028 (PMC3468530; doi:10.1371/journal.pone.0047028)
Supplement: Figure S3 — Electronic density map of Sis Lac at 2.7 Å resolution. (DOC) [file pone.0047028.s003.doc]

**
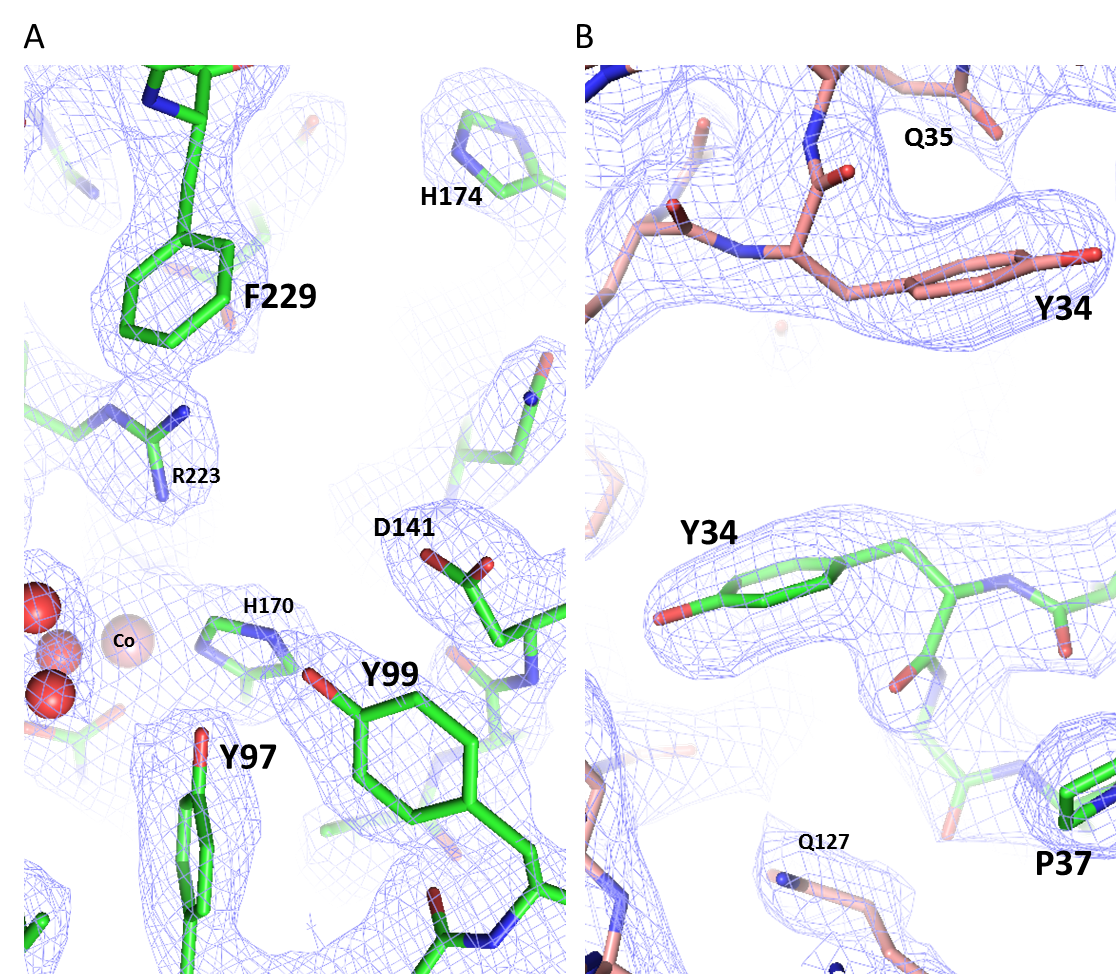
**

**Figure S3: Electronic density map of *Sis*Lac at 2.7 Å resolution**

Electronic density map (2F_obs_ – F_calc_) of *Sis*Lac at 2.7 Å resolution contoured at 1σ in the region of the active site (**A**) and Q34 (**B**). In panel **B.**, each monomer is represented in different color.
