# Supplementary material for: Structural and Enzymatic characterization of the lactonase SisLac from Sulfolobus islandicus
Source: PLoS One. 2012 Oct 10;7(10):e47028. doi: 10.1371/journal.pone.0047028 (PMC3468530; doi:10.1371/journal.pone.0047028)
Supplement: Figure S4 — Sequence alignment of PLLs from Sulfolobus species. (DOC) [file pone.0047028.s004.doc]

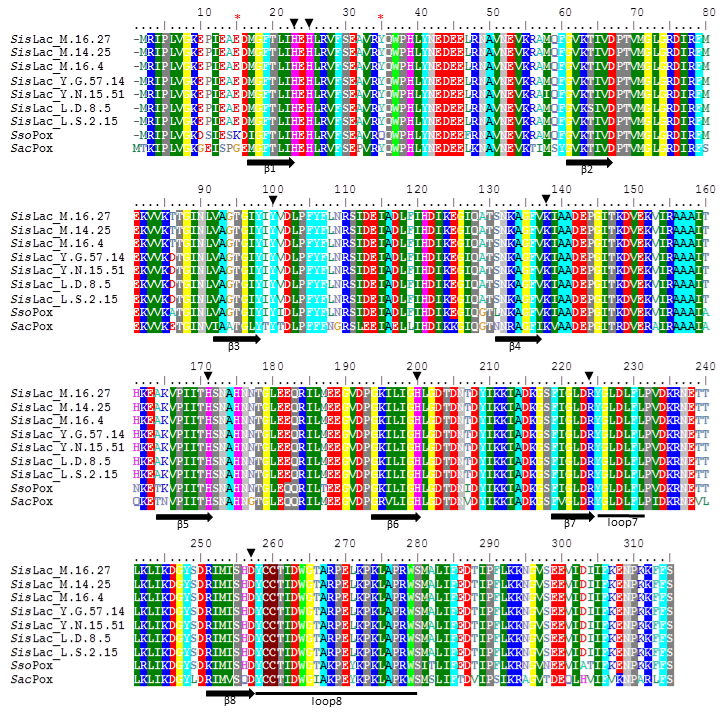


**Figure S4: Sequence alignment of PLLs from Sulfolobus species**

Sequence alignment of seven *Sis*Lac proteins (from strains ACR40964.1, ACP37197.1, ACP54338.1, ACP44606.1, ACP34489.1, ADB86112.1, ACP49818.1), *Sso*Pox from *S. solfataricus* strain MT4 (AAW47234.1), *Sac*Pox from *S. acidocaldarius* strain DSM 639 (AAY81433.1). Metal coordinating residues and important active site residues are marked by a black vertical arrow. The 8 β-sheets constituting the central barrel are indicated by a horizontal black arrow. Residues Y34 and K14 (according to the numerotation of *Sis*Lac_M.16.4) are indicated by a red star.
