# Supplementary material for: Structural and Enzymatic characterization of the lactonase SisLac from Sulfolobus islandicus
Source: PLoS One. 2012 Oct 10;7(10):e47028. doi: 10.1371/journal.pone.0047028 (PMC3468530; doi:10.1371/journal.pone.0047028)
Supplement: Figure S5 — Thermostability analysis of Sis Lac by circular dichroism. (DOC) [file pone.0047028.s005.doc]

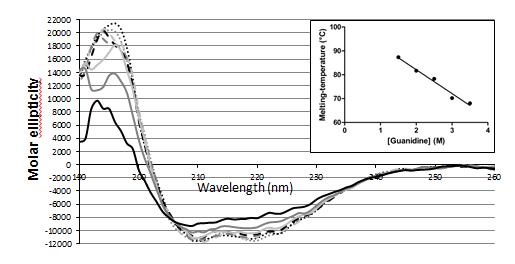


**Figure S5: Thermostability analysis of *Sis*Lac by circular dichroism**

Circular dichroism (CD) spectra of *Sis*Lac at different temperatures. The CD profile of *Sis*Pox at temperature 20 °C (*grey dotted line*), 30 °C (*black dotted line*), 40 °C (*grey dashed line*), 50 °C (*dark grey dashed line*), 60 °C (*black dashed line*), 70 °C (*grey continuous line*), 80 °C (*dark grey continuous line*) and 90 °C (*dark continuous line*) is represented without guanidinum chloride. The molar elypticity is plotted as a function of the wavelength over the range of 190-260 nm. In the right top corner is represented the linear regression of the *Sis*Lac T_m_ *versus* guanidinium chloride concentration which allow to extrapolate the T_m_ of the protein at 102 ± 2 °C at the y-intercept.
