# Supplementary material for: Structural and Enzymatic characterization of the lactonase SisLac from Sulfolobus islandicus
Source: PLoS One. 2012 Oct 10;7(10):e47028. doi: 10.1371/journal.pone.0047028 (PMC3468530; doi:10.1371/journal.pone.0047028)
Supplement: Figure S6 — Sis Lac metal preference. (DOC) [file pone.0047028.s006.doc]

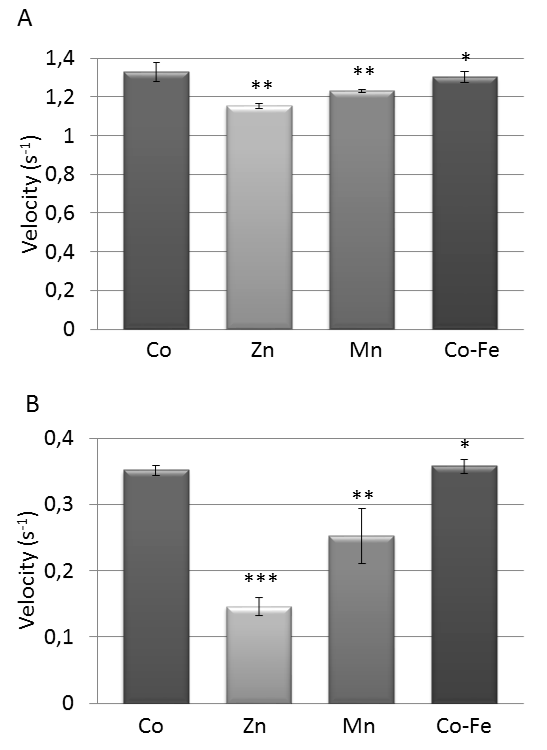


**Figure S6: *Sis*Lac metal preference**

**A**-**B**-Metal dependence of the paraoxonase (**A**) and lactone (**B**) activity. Velocity of *Sis*Lac against undecanoic-γ-lactone (5 mM) (*panel* ***A***) and ethyl-paraoxon (5 mM) (*panel* ***B***) in function of metal ion present in the buffer. Student test was performed between value obtained for cobalt ion and others: * *p* value > 0.05; ** *p* value < 0.05; *** *p* value < 0.001.
