# Supplementary material for: Structural and Enzymatic characterization of the lactonase SisLac from Sulfolobus islandicus
Source: PLoS One. 2012 Oct 10;7(10):e47028. doi: 10.1371/journal.pone.0047028 (PMC3468530; doi:10.1371/journal.pone.0047028)
Supplement: Information S1 — Supplementary information for Sis Lac metal preferences. (DOC) [file pone.0047028.s007.doc]

**Supplementary information for *Sis*Lac metal preferences**

***Materials & methods***

The effects of various divalent metal cations on *Sis*Lac lactonase (5 mM undecanoic-γ-lactone) and phosphotriesterase (5 mM ethyl-paraoxon) activity were measured. Lactonase activity was followed in *lactonase buffer* Bicine 2.5 mM, NaCl 150 mM, Cresol Purple 0.25 mM and the paraoxonase activity was assayed in buffer HEPES 50 mM pH 8, NaCl 150 mM. Different divalent cations were added to previous buffers: 0.2 mM CoCl_2_, ZnCl_2_, MnCl_2_ or 0.2 mM CoCl_2_ and 0.2 mM FeCl_2_. Aliquots of *Sis*Lac were diluted in each buffer (respectively 50 and 40 times dilution for lactonase and phosphotrieserase activity) and incubated for one night at 4 °C for substituting active site metals. The active site metals were thus exchanged by competition with various divalent cations. The lactonase and paraoxonase activity was respectively measured as previously explained using a microplate reader (Synergy HT) and Gen5.1 software in a 6.2 mm path length cell for 200 µL reaction in 96-well plate and at 25 °C. Each kinetic was performed in triplicate and Gen5.1 software and Excel software (Microsoft) allowed to evaluate the mean of the initial velocities of the protein for each metal and each activities.

***Results***

The metal dependency of *Sis*Lac was assayed using undecanoic-γ-lactone (**Fig. S3A**) and ethyl-paraoxon (**Fig. S3B**) as substrate. The experiments with zinc, manganese and cobalt show, for both activities, that *Sis*Lac exhibits the highest velocity with a cobalt ion as cofactor. This preference has also been seen for PTEs and for *Sso*Pox’s phosphotiresterase activities. *Dr*OPH exhibits the highest lactonase activity with cobalt or manganese as cofactor , MCP with manganese and *Gk*L only with zinc ion . The PLL and PTE families possess a buried and an exposed metal binding site. The buried site is likely to be an iron cation as seen by anomalous acattering on crystals in *Sso*Pox and PTE . However, the mixture iron/cobalt was assayed with *Sis*Lac, but showed no marked difference relative to the assay in cobalt-containing buffers.

1. Jackson CJ, Carr PD, Kim HK, Liu JW, Herrald P, et al. (2006) Anomalous scattering analysis of Agrobacterium radiobacter phosphotriesterase: the prominent role of iron in the heterobinuclear active site. Biochem J 397: 501-508.

2. Rochu D, Viguie N, Renault F, Crouzier D, Froment MT, et al. (2004) Contribution of the active-site metal cation to the catalytic activity and to the conformational stability of phosphotriesterase: temperature- and pH-dependence. Biochem J 380: 627-633.

3. Merone L, Mandrich L, Rossi M, Manco G (2005) A thermostable phosphotriesterase from the archaeon Sulfolobus solfataricus: cloning, overexpression and properties. Extremophiles 9: 297-305.

4. Xiang DF, Kolb P, Fedorov AA, Meier MM, Fedorov LV, et al. (2009) Functional annotation and three-dimensional structure of Dr0930 from Deinococcus radiodurans, a close relative of phosphotriesterase in the amidohydrolase superfamily. Biochemistry 48: 2237-2247.

5. Chow JY, Wu L, Yew WS (2009) Directed evolution of a quorum-quenching lactonase from Mycobacterium avium subsp. paratuberculosis K-10 in the amidohydrolase superfamily. Biochemistry 48: 4344-4353.

6. Chow JY, Xue B, Lee KH, Tung A, Wu L, et al. (2010) Directed evolution of a thermostable quorum-quenching lactonase from the amidohydrolase superfamily. J Biol Chem 285: 40911-40920.

7. Elias M, Dupuy J, Merone L, Mandrich L, Porzio E, et al. (2008) Structural basis for natural lactonase and promiscuous phosphotriesterase activities. J Mol Biol 379: 1017-1028.
