# Supplementary material for: Structural and Enzymatic characterization of the lactonase SisLac from Sulfolobus islandicus
Source: PLoS One. 2012 Oct 10;7(10):e47028. doi: 10.1371/journal.pone.0047028 (PMC3468530; doi:10.1371/journal.pone.0047028)
Supplement: Table S1 — Primers used for site directed mutagenesis. (DOC) [file pone.0047028.s008.doc]

**Table S1**: Primers used for site directed mutagenesis

| ***Sso*Pox-Q34Y-fw** | GCGAAGCAGTTCGT**TAT**CAGTGGCCTCATC |
| --- | --- |
| ***Sso*Pox-Q34Y-rev** | GATGAGGCCACTG**ATA**ACGAACTGCTTCGC |
| ***Sis*Lac-E14K-fw** | GTAAAGAACCGATTGAAGCC**AAA**GATATGGGTTTTACCCTG |
| ***Sis*Lac-E14K-rv** | CAGGGTAAAACCCATATC**TTT**GGCTTCAATCGGTTCTTTAC |
| ***Sis*Lac-Y34H-fw** | GCGAAGCAGTTCGT**CAT**CAGTGGCCTCATC |
| ***Sis*Lac-Y34H-rv** | GATGAGGCCACTG**ATG**ACGAACTGCTTCGC |
| ***Sis*Lac-Y34Q-fw** | GCGAAGCAGTTCGT**CAG**CAGTGGCCTCATCTG |
| ***Sis*Lac-Y34Q-rv** | CAGATGAGGCCACTG**CTG**ACGAACTGCTTCGC |

Underscored-bold nucleotides represent the sit directed mutagenesis point.
