# Supplementary material for: Structural and Enzymatic characterization of the lactonase SisLac from Sulfolobus islandicus
Source: PLoS One. 2012 Oct 10;7(10):e47028. doi: 10.1371/journal.pone.0047028 (PMC3468530; doi:10.1371/journal.pone.0047028)
Supplement: Table S2 — Kinetics protocols. (DOC) [file pone.0047028.s009.doc]

**Table S2: Kinetics protocols**

| **Type** | **Substrat** | **chromophore** | **Solvant** | **λ (nm)** | **ε (M^-1^.cm^-1^)** | **concentration range** |
| --- | --- | --- | --- | --- | --- | --- |
| **Phosphotriesters** | Paraoxon | *p*NP | Ethanol | 405 | 17 000 | 50-18 000 µM |
|  | Parathion | *p*NP | Ethanol | 405 | 17 000 | 50-1 000 µM |
|  | Malathion | DTNB | Ethanol/DMSO | 412 | 13 700 | 50-2 000 µM |
|  | Coumarin nerve agent derivative (CMP) | cyanocoumarin | methanol | 412 | 37 000 | 10-750 µM |
| **Esters** | Phenyl-acetate | Phenolate | Ethanol | 270 | 1 400 | 500 µM |
|  | *p*NP-acetate | *p*NP | Ethanol | 405 | 17 000 | 50-5 000µM |
|  | *p*NP-Decanoate | *p*NP | Ethanol | 405 | 17 000 | 100 µM |
|  | *m*NP-Acetate | *m*NP | Ethanol | 408 | 1 680 | 450 µM |
|  | 4-acetoxy-acetophenone | acetophenone | DMSO | 320 | 16 160 | 100 µM |
| **Lactones** | di-hydrocoumarin | Coumarin | DMSO | 270 | 1400 | 50-5 000 µM |
|  | Lactones | Cresol purple | DMSO | 577 | 1 837-5 517^a^ | 50 -2 000 µM |

^a^ calculated as explained in method.

1. Hong SB, Raushel FM (1996) Metal-substrate interactions facilitate the catalytic activity of the bacterial phosphotriesterase. Biochemistry 35: 10904-10912.

2. Ellman GL, Courtney KD, Andres V, Jr., Feather-Stone RM (1961) A new and rapid colorimetric determination of acetylcholinesterase activity. Biochem Pharmacol 7: 88-95.

3. Ashani Y, Gupta RD, Goldsmith M, Silman I, Sussman JL, et al. (2010) Stereo-specific synthesis of analogs of nerve agents and their utilization for selection and characterization of paraoxonase (PON1) catalytic scavengers. Chem Biol Interact 187: 362-369.

4. Khersonsky O, Tawfik DS (2005) Structure-reactivity studies of serum paraoxonase PON1 suggest that its native activity is lactonase. Biochemistry 44: 6371-6382.

5. Afriat L, Roodveldt C, Manco G, Tawfik DS (2006) The latent promiscuity of newly identified microbial lactonases is linked to a recently diverged phosphotriesterase. Biochemistry 45: 13677-13686.
