# Supplementary material for: Structural and Enzymatic characterization of the lactonase SisLac from Sulfolobus islandicus
Source: PLoS One. 2012 Oct 10;7(10):e47028. doi: 10.1371/journal.pone.0047028 (PMC3468530; doi:10.1371/journal.pone.0047028)
Supplement: Table S3 — Ethyl-paraoxonase comparison between Sso Pox, Sac Pox and Sis Lac. (DOC) [file pone.0047028.s010.doc]

**Table S3: Ethyl-paraoxonase comparison between *Sso*Pox, *Sac*Pox and *Sis***Lac

| **Conditions** | ***Sis*Lac** | | | ***Sso*Pox** | | | ***Sac*Pox^c^** | | |
| --- | --- | --- | --- | --- | --- | --- | --- | --- | --- |
|  | **k_cat_ (s^-1^)** | **K_M_ (µM)** | **k_cat_/K_M_ (s^-1^M^-1^)** | **k_cat_ (s^-1^)** | **K_M_ (µM)** | **k_cat_/K_M_ (s^-1^M^-1^)** | **k_cat_ (s^-1^)** | **K_M_ (µM)** | **k_cat_/K_M_ (s^-1^M^-1^)** |
| **25 °C** | 1.42 ± 0.09 | 5439 ± 873 | 2.60(±0.58)x10^2^ | - | - | - | - | - | - |
| **25 °C SDS 0.1%** | 14.31 ± 3.16 | 2005 ± 728 | 7.14(±4.16)x10^3^ | - | - | - | - | - | - |
| **25 °C SDS 0.01%** | 2.70 ± 0.29 | 4248 ± 999 | 6.36(±2.18)x10^2^ | - | - | - | - | - | - |
| **70 °C** | 0.79 ± 0.04 | 1131 ± 196 | 6.98(±1.56)x10^2^ | 0.24 ± 0.01^a^ | 60 ± 9^a^ | 4.00(±0.75)x10^3a^ | ND | ND | 0.91 ± 0.06 |
|  |  |  |  | 2.29^b^ | 2360^b^ | 9.7x10^2b^ |  |  |  |
|  |  |  |  |  |  |  |  |  |  |

^a^ from Merone *et. al* (2005); ^b^ from Ng *et al*. (2010); ^c^ from Porzio *et al.* (2007), data were obtained at 75 °C. Data obtained with cobalt as cofactor. – corresponds to not tested substrates and ND corresponds to not determined values.
