# Supplementary material for: Structural and Enzymatic characterization of the lactonase SisLac from Sulfolobus islandicus
Source: PLoS One. 2012 Oct 10;7(10):e47028. doi: 10.1371/journal.pone.0047028 (PMC3468530; doi:10.1371/journal.pone.0047028)
Supplement: Table S4 — Phosphotriesterase activity comparison between Gs P, Dr OPH and Sso Pox and Sis Lac. (DOC) [file pone.0047028.s011.doc]

**Table S4: Phosphotriesterase activity comparison between *Gs*P, *Dr*OPH and *Sis***Lac

| **Substrate** | | ***Sis*Lac** | | | ***Gs*P^a^** | | | ***Dr*OPH^b^** | | |
| --- | --- | --- | --- | --- | --- | --- | --- | --- | --- | --- |
| **Name** | **Structure** | **k_cat_ (s^-1^)** | **K_M_ (µM)** | **k_cat_/K_M_ (s^-1^M^-1^)** | **k_cat_ (s^-1^)** | **K_M_ (µM)** | **k_cat_/K_M_ (s^-1^M^-1^)** | **k_cat_ (s^-1^)** | **K_M_ (µM)** | **k_cat_/K_M_ (s^-1^M^-1^)** |
| **Ethyl-Paraoxon** | 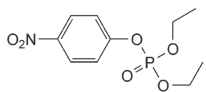 | 1.42 ± 0.09 | 5 439 ± 873 | 2.6(±0.58)x10^2^ | 0.12 ± 0.02 | 2100 ± 320 | 5,47(±0.47)x10^1^ | 4.16 (±0.13)x10^-3^ | 3 000 ± 200 | 1.39 ± 0.11 |
| **Methyl-Paraoxon** | 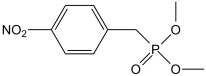 | 7.40 ± 1.26 | 1 739 ± 417 | 4.26(±1.74)x10^3^ | 7.83(±0.33)x10^-3^ | 270 ± 31 | 2.90(±0.13)x10^1^ | 7.67(±0.33)x10^-4^ | 1300 ± 100 | 0.58 ± 0.05 |

^a^ from Hawwa *et al*. (2009), data characterized at 35 °C. ^b^ from Hawwa *et al*. (2009). Data obtained with cobalt as cofactor.
